# Highlighting the Importance of Water Alkalinity Using Phosphate Buffer Diluted With Deionized, Double Distilled and Tap Water, in Lowering Oxidation Effects on Human Hemoglobin Ozonated at High Ozone Concentrations *in vitro*

**DOI:** 10.3389/fmolb.2020.543960

**Published:** 2020-10-08

**Authors:** Fouad Mehraban, Saeed Rayati, Vahid Mirzaaghaei, Arefeh Seyedarabi

**Affiliations:** ^1^Institute of Biochemistry and Biophysics, University of Tehran, Tehran, Iran; ^2^Department of Chemistry, K. N. Toosi University of Technology, Tehran, Iran; ^3^The Founder of Gardina Corporation and Manufacturer of Ozone Therapy Devices in Tehran, Tehran, Iran

**Keywords:** human hemoglobin, phosphate buffer, various water types, bicarbonate ions, water alkalinity, ozone, oxidation, autohemotherapy

## Abstract

In autohemotherapy, it is important to find a way to lower the effects of oxidation, especially at high concentrations of ozone. One of the parameters, other than ozone concentration, which can have a significant effect on the stability and rate of decomposition of ozone at high concentrations, is the presence of ions in water. A number of spectroscopic techniques including intrinsic fluorescence, circular dichroism and UV–VIS were used as well as SDS-PAGE, Native-PAGE dynamic light scattering and water ion analysis, in order to investigate the effects of two relatively high concentrations of ozone on purified human hemoglobin (Hb) in phosphate buffer and diluted versions with deionized, double distilled and tap water *in vitro*. Purified human Hb and not whole blood human Hb was used in this study, since the addition of water to the whole blood would have caused the RBCs to lyse, affecting the purification of Hb for further analysis. Therefore, using purified Hb, it was possible to investigate the effects of dilution of 50 mM phosphate buffer to 10 mM phosphate buffer with different water types including non-ion containing deionized and double distilled water as well as ion-containing tap water, when ozonated at 55 and 80 μg/ml ozone. The fundamental changes in the secondary and tertiary structures of Hb were seen to be related to non-ozonated Hb samples diluted with deionized and double distilled waters, respectively. Generally, Hb oligomerization was more likely to occur at the higher concentration of ozone (80 μg/ml) and in samples where phosphate buffer was diluted with non-ion containing deionized and double distilled waters and not the ion-containing tap water. This could be explained by the presence of water alkalinity or bicarbonate ions in tap water, which can scavenge free radicals and reduce Hb oxidation/oligomerization. Therefore, it was concluded that Hb could best withstand high concentrations of ozone in the presence of the undiluted 50 mM phosphate buffer followed by phosphate buffer diluted with tap water, containing bicarbonate ions.

## Introduction

Water molecules have a special and comprehensive role in biology. The folding, stability and function of protein are closely related to their interactions with water molecules (Royer et al., 1996). It has been shown that ion hydration plays a very important role in the biological effects of ions. Biological evidence has also shown that the short-range nature of the main forces are created by ions in water (Marcus, 2009). Internal waters are classified into three categories: (1) water that binds metal ions, (2) water that binds to charged protein groups, and (3) water that binds polar groups. Each type of water molecules listed help to stabilize the protein structure. In many of the top conditions that water can have, water plays an electrostatic role and reduces the effective electrostatic potential of the internal charges by increasing the local di-electric and binding charges in order to form ion pairs separated by water. As a result, water can stabilize metal ions, charged groups and inappropriate dipoles. The internal waters are clusters of one to several molecules in various protein, and often coordination numbers are three to four, and preferably, are hydrogen donors. In accordance with the mentioned topic, internal waters bond 1.9 times more to the protein main chain carbonyls (CO) than the nitrogen main chain (NH), which reflects the various capacities of the carbonyl and amide nitrogen groups (almost at a ratio of 2 carbonyl:1 amide nitrogen group) in hydrogen bond formation. It is reported that for internal and external waters together, this ratio is 2.8 to 1. Additionally, a study has shown that aspartic acid and glutamic acid (Asp and Glu) are both responsible for 35% of side-chain hydrogen bonds with internal waters (Teeter, 1991).

Ozone has high oxidant potency (Elvis and Ekta, 2011) and ozone therapy belongs to the category of bio-oxidative therapies and its therapeutic effects have been investigated in various diseases (Bocci et al., 2009). Ozone acts based on the theory of hormesis (oxidative preconditioning) (Leon et al., 1998; Calabrese, 2010; Sagai and Bocci, 2011; Re et al., 2014), in which repeated use of calculated and appropriate concentrations of ozone (lower than toxic and inhibitory concentrations) in the correct way associated with the type of disease can have beneficial therapeutic effects (Bocci et al., 2007; Sagai and Bocci, 2011; Smith et al., 2015). According to the hormesis theory, ozone can have its therapeutic effects by producing the mild and controlled oxidative stress by reacting with biological molecules in the blood circulation (Sagai and Bocci, 2011).

Autohemotherapy is the most common method of treatment using ozone, in which the specific amount of blood in the presence of sodium citrate (an anticoagulant), in the form of *ex vivo*, is mixed with the same amount of ozone gas mixture (oxygen 96–99% plus 4-1% ozone, respectively) at a specified concentration (Viebahn-Hansler et al., 2012; Smith et al., 2015). In this case, ozone generates precise, mild and transient oxidative stress, while endogenous oxidative stress is more durable and lasting (Bocci et al., 2011a). Studies have shown that ozone with low and calculated concentrations exerts its beneficial effects such as reducing endogenous oxidative stress through an adaptive response (Bocci, 2006, 2007; Bocci et al., 2009).

Ozone dissolves rapidly in the aquatic environment of the blood and gives its energy to the most hematic portion of the blood, hemoglobin (Hb) (Bocci et al., 2011b). Recently, the effects of ozone on Hb have been extensively studied (Karnaukhova et al., 2014; Mehraban et al., 2018, 2019).

It has been reported that ozone has a half-life in the range of seconds to hours depending on the quality of water (Von Gunten, 2003). Furthermore, ozone stability mainly depends on the water matrix, especially its pH and alkalinity (Hoigne, 1998). The role of pH of water is related to hydroxide ions at the onset of the ozone decomposition process (Von Gunten, 2003) and the main secondary oxidant formed by the decomposition of ozone in water is OH radicals (Hoigne, 1998). Ozone has been shown to be unstable in aqueous conditions and its decomposition depends on the nature of the water quality and its pH. Hydroxide ions in the dominant chain reaction initiate ozone decomposition. Ozone can be decomposed faster either by increasing pH or adding H_2_O_2_ (Sehested et al., 1998; Von Gunten, 2003; Sharma and Graham, 2010).

Additionally, it has been determined that the chemical effects of ozone in water and its decomposition rate, as well as the secondary oxidants formed during ozonation, depends on the type of water and its chemical composition, as well as the concentration of ozone (Hoigne, 1994; Hoigne and Bader, 1994). The chemical composition of water in ozonation is a significant factor that significantly affects the lifetime of ozone and thus the amount of ozone transferred from the gas state to the liquid state, the actual concentration of ozone imported during the reaction time, the ozone remaining at the end of the ozonation process and the formation of secondary oxidants such as OH radicals. The chemical composition of water also affects the production of by-products of ozonation and the formation and consumption of secondary oxidants (Hoigne, 1994). The pH value and the concentration of inorganic and organic molecules in water, which determine the changes in the chemical composition of water, can affect all aspects related to ozonation, and even the half-life of ozone, which may vary in magnitude (Hoigne and Bader, 1979).

Carbonate alkalinity has been shown to have a great effect on the stability of ozone. In fact, carbonate and bicarbonate ions act as inhibitors of the ozone decomposition cycle as these ions scavenge OH radicals so that they cannot produce superoxides and any other species that accelerates ozone decomposition. Therefore, increment in carbonate alkalinity results in more inhibition and reduction of the rate of ozone decomposition (Elovitz et al., 2000). Ions such as carbonate and bicarbonate can increase ozone half-life by scavenging OH radicals that can decompose ozone. Dissolved organic matter from different pathways can interfere with ozonation. Nitrites, iron, chloride and manganese may also be effective in the use and consumption of ozone (Hoigne, 1994).

Ozone can increase the concentration of oxygenated functional groups such as carboxylic acid and lead to interaction with coagulation cations or natural available cations such as calcium in water hardness. Ozone dose/concentration, coagulation conditions (coagulant type and dose) and properties of raw water (such as hardness and alkalinity) as important indicators, determine the effect of ozone on coagulation (Bose and Reckhow, 2007). It is reported that ozonation, prior to coagulation, is useful for waters with moderate to high levels of calcium hardness. Water particle aggregation by ozone occurs only in the presence of significant concentrations of calcium (Chandrakanth and Amy, 1996). It has been reported that when calcium concentrations in raw water are more than 100 mg/Lit CaCO_3_, ozone can increase coagulation (Chandrakanth, 1994). It has also been shown that ozone reduces the stability of water particles and the concentration of calcium. These simultaneous effects of ozone can improve the coagulation process and increase the removal of water particles, which ultimately leads to a reduction in coagulant concentration when ozone concentrations are increased (Sadrnourmohamadi and Gorczyca, 2015).

Furthermore, studies have shown that chloride ions (Cl^–^) significantly accelerate the decomposition of ozone in water (Razumovskii et al., 2010) and can destroy stratospheric ozone (Mu et al., 2000). It is also known that sulfate can cause stratospheric ozone loss (Tabazadeh et al., 2002).

Structural changes in protein in the presence of multiple compounds can be explained by their respective interactions (Kamshad et al., 2019; Mokaberi et al., 2019, 2020). In Hb, structural changes have been reported upon ozonation in different environments and under different conditions using multiple spectroscopic techniques (Mehraban et al., 2018, 2019).

The goal of this study was to determine the role of ions in different types of water using phosphate buffer diluted with deionized, double distilled and tap water on purified human Hb at two relatively high concentrations of ozone. Based on our goal, we investigated and compared, for the first time, the effects of ozone at 55 and 80 μg/ml on purified human Hb of a healthy individual in the presence of 50 mM phosphate buffer (pH 7.4) prepared with double distilled water (dd water) and diluted versions (10 mM phosphate buffer) with various water types including again double distilled water, deionized water (dio water), and drinkable tap water (tap water). Part of the goal was also to determine what type of water or more specifically, the ions needed to withstand the damage which can be caused by high concentrations of ozone, as high as 80 μg/ml, on human Hb. Purified human Hb and not whole blood Hb was used in this study, since the addition of water to the whole blood would have caused the red blood cells (RBCs) to lyse (Krzyzaniak et al., 1997), thus affecting the purification of Hb (Andrade et al., 2004) for further analysis. This study was performed using various techniques including intrinsic fluorescence, CD, and UV-VIS spectroscopies, in order to examine the changes in the secondary and tertiary structure of Hb and its heme group and aromatic residues, as well Native- and SDS-PAGE, in the presence and absence of dithiothreitol (DTT) (as a reducing agent) and dynamic light scattering (DLS) to analyze the oligomerization and polydispersity of Hb upon exposure to the two different concentrations of ozone in the presence of phosphate buffer and diluted versions with different water types. Additionally, phosphate buffer and the different water types were assessed by a conductivity meter to measure their conductivity and hence the presence or absence of different ions.

## Materials and Methods

### Water Ion Analysis Using the Conductivity Measurement Test

A Jenway model 4520 conductivity meter fitted with a glass *K* = 1 conductivity cell was used to take conductivity measurements. Alkalinity was determined using the titrimetric method with H_2_SO_4_ 0.02 N and phenolphthalein and methyl orange as indicators. For measuring total hardness, a complexometric titration method using EDTA 0.02 N was applied. An ammonia/ammonium chloride buffer solution and eriochrome black T were used for pH adjustment of the water sample at 10.0 and indicator, respectively. The permanent hardness was calculated by subtracting the alkalinity from the total hardness. An argentometric titration using Mohr’s method was used for determination of chloride concentration (mg/Lit CaCO_3_) in the water sample. Moreover, for measuring the sulfate ion concentration, an extra amount of barium chloride was added in the water sample. Then, the remaining barium ion concentration was determined using a complexometric titration method with EDTA. The sulfate concentration (mg/Lit CaCO_3_) was calculated by subtracting the excess barium from the total amount.

### Ozone Generation

Ozone was generated by passing pure oxygen gas using an electrical corona arc discharge and by a generator made by Gardina Corporation (Iran). The flow and concentration of ozone gas was controlled by photometry (Mehraban et al., 2018). The use of filtered air due to the presence of 78% nitrogen results in the formation of nitrogen oxides which is not desirable in ozone therapy. Ozone-resistant syringes were used to stabilize the concentration and prevent ozone leakage and contamination.

### Collection and Preparation of Hb

Blood was first taken from a healthy person and then Hb purified from it similar to our previous study (Mehraban et al., 2018). In summary, after centrifugation and removal of plasma, the red blood cells were washed with saline and lysed with cold water, followed by addition of ammonium sulfate to precipitate and separate the redundant protein before Hb was dialyzed in phosphate buffer. The dialyzed Hb was then frozen until use.

### Gas Delivery and Sample Treatments

In all of the experiments, a single concentration of ozone gas mixture with a flow rate of 0.8 L/min, containing 95–99% oxygen plus 5–1% ozone, respectively, expressed in μg/ml (concentration per volume) was used. The final pressure of ozone was kept at normal atmospheric pressure (Travagli et al., 2007). In major autohemotherapy (O_3_-AHT), the therapeutic concentration of ozone for various diseases varies from low to maximal 80 μg/ml, depending on the type of disease (Bocci V. A. et al., 2011). The best duration for mixing ozone with blood is 5 min (Viebahn-Hansler et al., 2012), which is the time required to achieve a complete and homogeneous equilibrium of viscous blood with ozone (Bocci, 2005).

As mentioned previously, adding water to red blood cells (RBC) in whole blood causes the RBCs to lyse (Krzyzaniak et al., 1997) and affects the purification of Hb (Andrade et al., 2004) for further analysis. Therefore, in this study, in order to simulate the autohemotherapy conditions and to prevent the RBCs from lysing upon addition of different water types prior to purification of Hb and to investigate the effects of different ions in ozonation of Hb, purified Hb samples from a healthy individual’s whole blood was ozonated in the presence of phosphate buffer (pH ∼7.4) and diluted versions with added deionized water (dio), double distilled water (dd) and tap water. Purified Hb was exposed to ozone gas in a 1:1 volumetric ratio and treated with two concentrations of 55 and 80 μg/ml ozone, for 5 min. The control blood samples were not exposed to ozone.

### Fluorescence Spectroscopy

The fluorescence spectra were measured, as in our previous study (Mehraban et al., 2018), using excitation at a wavelength of 280 nm and emission in the range of 300 to 400 nm. Protein concentration was determined from standard absorbance measurements using a UV spectrophotometer at 280 nm. The bandwidths of the monochromator for excitation and emission were both set at 10 nm. The cuvette cell path length was 1 cm and the concentration of the samples was 5 mg/ml.

### CD Measurements

All CD tests, similar to our previous study (Mehraban et al., 2018) were performed using an AVIV 215 spectropolarimeter (Aviv Associates, Lakewood, NJ, United States) and the results were plotted as ellipticity (in deg. cm^2^ dmol^–1^) versus wavelength in nanometers. Protein concentration of samples for Far-UV CD and Near- and Soret-UV CD measurements were 0.2 mg/ml and 0.5 mg/ml, respectively. The cuvette cell path lengths for Far-UV CD and Near- and Soret-UV CD measurements were 0.1 and 1 cm, respectively. The number of repeated consecutive scans for Far-UV and Near- and Soret-UV CD were 1 and 4, respectively.

### UV-VIS Absorption Scan Spectroscopy

The UV-visible spectrophotometer (Varian, Carry 100 Bio, Australia) was used to investigate the absorbance scanning spectrum at a wavelength of 280 nm and in the range of 200 to 700 nm. The cuvette cell path length was 1 cm and the concentration of the samples was 5 mg/ml.

### SDS-PAGE and Native-PAGE Electrophoresis

At all stages, SDS-PAGE and Native-PAGE studies were performed using 18% gel and the molecular weights of the Hb samples were compared with the protein marker (SMOBiO, Taiwan). Hb at 20 mg/ml was prepared with phosphate buffer and diluted with various water types to assess the formation of oligomers at high ozone concentrations.

### Dynamic Light Scattering

To measure the DLS of Hb samples, the same method was used as reported in our previous study (Mehraban et al., 2019). The refractive index of the material (protein) and the absorption were set to 1.59 and 0.01, respectively. The following parameters were used: the dispersant viscosity was 0.8872 cP and the refractive index was 1.330. The particle size obtained by the DLS method is not the actual particle size, but the hydrodynamic size (Panalytical, 2010).

### Statistical Analysis

The results were averages of three measurements and reported as means ± SD using a non-parametric Friedman test and presented by the SPSS software, version 18.

## Results

### Conductivity Measurements for Phosphate Buffer and Various Water Samples

The results of water analysis are summarized in Table 1. Neither the double distilled water nor the deionized water gave any readings. Only the phosphate buffer and tap water had conductivity measurements as reported in Table 1, showing the presence of ions. The EC value showing the concentration of purely phosphate ions for the undiluted phosphate buffer was 4.4 μS/cm. As for tap water, this value was 0.2 μS/cm, consisting of bicarbonate, calcium, magnesium, chloride and sulfate ions.

**TABLE 1:** Analysis of various parameters of phosphate buffer and tap water.

Parameters	Phosphate buffer	Tap water
EC (μS/cm)	4.4	0.2
Total Hardness (mg/Lit CaCO_3_)	–	170
Temporary Hardness (mg/Lit CaCO_3_)	–	113
Permanent Hardness (mg/Lit CaCO_3_)	–	57
Alkalinity (mg/Lit CaCO_3_)	–	113
Chloride (mg/Lit CaCO_3_)	–	50
Sulfate (mg/Lit CaCO_3_)	–	90

*EC measurement shows the total amount of ions present. Hardness measurements of water refers to the presence of calcium and magnesium ions, whereas, alkalinity measurements show the existence of bicarbonate ions.*

The presence of bicarbonate ions in tap water can be useful in inhibiting ozone decomposition, which would otherwise lead to the production of harmful superoxides. Additionally, it can increase the half-life of ozone for a more moderate and long term effect on Hb and prevent sudden and harsh levels of oxidation, which may lead to oligomerization via disulfide and di-tyrosine cross linking, especially at higher concentrations of ozone. On the contrary, the presence of ions such as calcium in tap water can result in coagulation of carboxylic groups of Hb residues with calcium ions during ozonation. Furthermore, the existence of chloride and sulfate ions in tap water, which are involved in the decomposition of ozone, can increase the level of superoxides in solution and therefore have adverse effects on ozonated Hb in terms of oligomerization.

### pH Variations of Non-ozonated and Ozonated Hb in Phosphate Buffer and Diluted Versions With Different Water Types

Table 2 shows pH changes related to ozonation in phosphate buffer and diluted versions with different water types. Due to the fact that phosphate buffer was diluted with different water types and was present in all samples, a tampon effect of phosphate buffer was seen, such that no severe pH changes, even at higher concentrations of ozone was detectable. Therefore, dilution of phosphate buffer from 50 to 10 mM in the presence of different waters did not make a significant change in the pH. Only in the case of tap water, relative to the rest, due to various compounds and ions (as shown in Table 1), a slight increase in pH was observed, which may play a role in a slight increase in the speed of decomposition of ozone (Von Gunten, 2003). Interestingly, the coagulation between carboxylic groups of acidic residues such as aspartic and glutamic acids in Hb upon ozonation in the presence of calcium (in tap water; defined as the hardness) can increase the pH and hence the decomposition of ozone, which leads again to the disadvantageous production of hydroxyl radicals (Hoigne, 1998). Having said that, the presence of bicarbonate ions in tap water reduces the ozone decomposition at the same time.

**TABLE 2:** Investigating pH changes of non-ozonated and ozonated Hb samples in the presence of phosphate buffer and diluted versions with different water types.

Hb buffer Or various water types	pH of Non-ozonated Hb	pH of Hb ozonated at 55 μg/ml	pH of Hb ozonated at 80 μg/ml
Phosphate buffer	7.46	7.49	7.47
Dio water	7.46	7.46	7.47
DD water	7.44	7.42	7.43
Tap water	7.60	7.51	7.51

*The pH values are averages of three readings.*

### The Effect of Pressure on Hb in Ozonation

**FIGURE 1:**
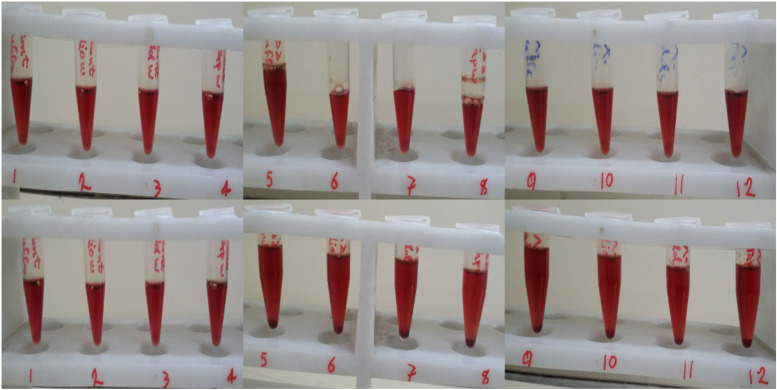
The effect of syringe volume on human Hb precipitation during ozonation. Top row: Hb ozonated at 55 μg/ml ozone (samples 5 to 8) and 80 μg/ml of ozone (samples 9 to 12) with the larger 10 ml syringe. Bottom row: Hb ozonated at 55 μg/ml ozone (samples 5 to 8) and 80 μg/ml of ozone (samples 9 to 12) with the smaller 5 ml syringe. Samples 1 to 4, 5 to 8 and 9 to 12 are Hb in phosphate buffer, deionized water, double distilled water and tap water, respectively, whereas samples 1–4 are non-ozonated control samples.

In this study we also had a look at the use of different size syringes when mixing Hb with ozone for 5 min. Therefore, two different sized syringes were chosen; one 5 ml and the other 10 ml, with high and low pressures upon mixing, respectively. A fixed volume of 2 ml Hb (in phosphate buffer and diluted versions with different water types) was mixed with 2 ml of ozone gas at both 55 and 80 μg/ml concentrations in both syringes. In general, what we observed was that the increased pressure in the smaller syringe, in the process of ozonation of Hb in phosphate buffer and diluted versions of various water types, resulted in Hb precipitation, which was seen in all samples (Figure 1 bottom panel, samples 5–12).

### Fluorescence Analysis

Fluorescence is used to detect changes in the tertiary structure of protein such as Hb through its intrinsic fluorophores, i.e., aromatic residues (tyrosines, tryptophans and phenylalanines) (Munoz and De Juan, 2007), especially tryptophan 37 in the beta chain, which is located in the α1β2 interface and has a major role in the emergence of the fluorescence spectrum (Basu and Kumar, 2015).

**FIGURE 2:**
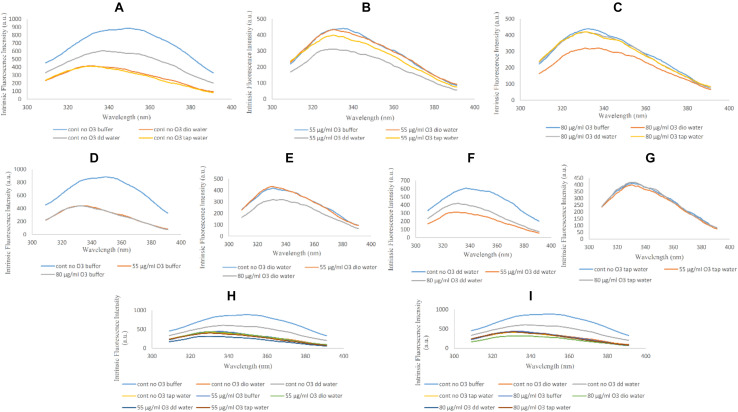
Intrinsic fluorescence spectra of non-ozonated and ozonated Hb samples at concentrations of 55 and 80 μg/ml in the presence of phosphate buffer and diluted versions with different water types. The fluorescence spectra were measured after excitation at 280 nm. **(A)** Non-ozonated Hb in phosphate buffer and different water types. **(B)** Hb ozonated with 55 μg/ml ozone in phosphate buffer and different water types. **(C)** Hb ozonated with 80 μg/ml ozone in phosphate buffer and different water types. **(D)** Non-ozonated and ozonated Hb with 55 and 80 μg/ml ozone in phosphate buffer. **(E)** Non-ozonated and ozonated Hb with 55 and 80 μg/ml ozone in phosphate buffer diluted with deionized (Dio) Water. **(F)** Non-ozonated and ozonated Hb with 55 and 80 μg/ml ozone in phosphate buffer diluted with double distilled (DD) Water. **(G)** Non-ozonated and ozonated Hb with 55 and 80 μg/ml ozone in phosphate buffer diluted with Tap Water. **(H)** Non-ozonated and ozonated Hb with 55 μg/ml ozone in phosphate buffer and diluted in various different water types. **(I)** Non-ozonated and ozonated Hb with 80 μg/ml ozone in phosphate buffer and diluted in various different water types.

The reduction in fluorescence peak intensity is indicative of a more open structure compared to the native non-ozonated form of Hb. Comparing non-ozonated Hb samples in the presence of phosphate buffer and diluted versions with different water types, it can be seen that Hb has the highest fluorescence peak intensity in non-diluted phosphate buffer, while Hb samples diluted with deionized water and tap water were the same, with the lowest peak intensities. The fluorescence peak intensity of the Hb sample with double distilled water had intermediate effects (Figure 2A). In relation to Hb samples ozonated at 55 μg/ml, the intensity of the fluorescence peak was reduced by almost half in all the samples (Figure 2B). The lowest peak intensity was related to double distilled water. Band intensity changes in other samples were not very significant. In Hb samples ozonated at 80 μg/ml, the intensity of the peaks were again reduced by almost a half as compared to the non-ozonated Hb samples, whereas Hb in deionized water had the lowest peak intensity, and Hb in other water types did not show any dramatic changes (Figure 2C).

The intensity of the fluorescence peak for the non-ozonated Hb sample in undiluted phosphate buffer revealed a two-fold increase compared to the two ozonated samples. In addition, ozonation of Hb caused the fluorescence peak to shift to the left and a shorter wavelength, giving a blue shift (Figure 2D). In Hb samples where the phosphate buffer was diluted with deionized water, upon comparing the effects of ozone, it was clear that Hb ozonated with 80 μg/ml ozone had a reduction in the fluorescence peak intensity compared to the non-ozonated Hb sample. However, Hb ozonated at 55 μg/ml had a similar peak intensity to non-ozonated Hb, higher than the peak intensity for Hb ozonated at 80 μg/ml ozone (Figure 2E). Hb samples in phosphate buffer diluted with double distilled water showed the most significant changes compared to the other water types. Control (non-ozonated) Hb sample in the presence of double distilled water had a higher fluorescence peak intensity, however, as already mentioned, had a lower peak intensity relative to the control Hb sample in the presence of undiluted phosphate buffer. Ozonation of Hb diluted with double distilled water decreased the intensity of the fluorescence peak, and ozonation at 55 μg/ml ozone showed more reduced peak intensity than 80 μg/ml (Figure 2F). There were no significant changes in the control (non-ozonated) and ozonated Hb samples in the presence of tap water (Figure 2G). From a general point of view, comparing the absence of ozone with the effects of ozone at the two different concentrations on Hb samples (Figures 2H,I), we found that the control (non-ozonated) Hb had the highest peak intensity in the presence of undiluted phosphate buffer followed by a major reduction in peak intensity in the control (non-ozonated) Hb in phosphate buffer diluted with double distilled water (from a concentration of 50 to 10 mM) and Hb samples diluted with different water types in the presence or absence of ozone.

### CD Measurements

#### Far-UV CD

The alteration of the secondary structure and content of alpha helix protein can be traced through the CD spectrum (Munoz and De Juan, 2007). This change in the structure of the alpha helix of protein can be seen as two strongly negative peaks at 209 and 222 nm. The first is related to the π-π^∗^ transition in α-helix and the second is the result of n-π^∗^ transition in both α-helix and unordered structure conformations (Li et al., 2014).

**FIGURE 3:**
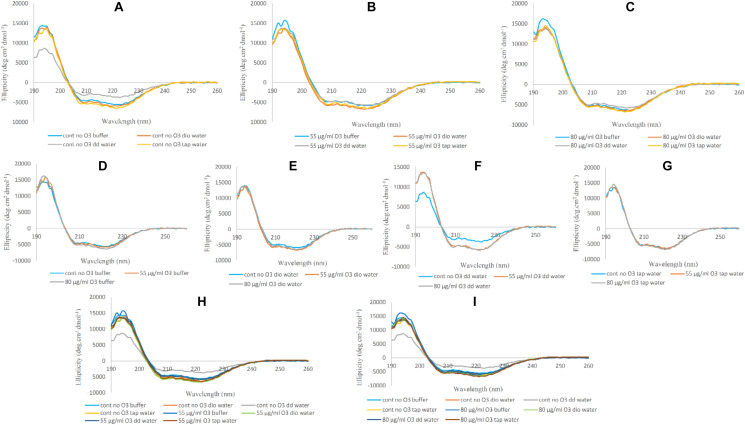
Far-UV CD spectra of non-ozonated and ozonated Hb samples in the presence of phosphate buffer and diluted versions with different water types. **(A)** Non-ozonated Hb in phosphate buffer and different water types. **(B)** Hb ozonated with 55 μg/ml ozone in phosphate buffer and different water types. **(C)** Hb ozonated with 80 μg/ml ozone in phosphate buffer and different water types. **(D)** Non-ozonated and ozonated Hb with 55 and 80 μg/ml ozone in phosphate buffer. **(E)** Non-ozonated and ozonated Hb with 55 and 80 μg/ml ozone in phosphate buffer diluted with deionized (Dio) Water. **(F)** Non-ozonated and ozonated Hb with 55 and 80 μg/ml ozone in phosphate buffer diluted with double distilled (DD) Water. **(G)** Non-ozonated and ozonated Hb with 55 and 80 μg/ml ozone in phosphate buffer diluted with Tap Water. **(H)** Non-ozonated and ozonated Hb with 55 μg/ml ozone in phosphate buffer and diluted in various different water types. **(I)** Non-ozonated and ozonated Hb with 80 μg/ml ozone in phosphate buffer and diluted in various different water types.

Changes in Hb Far-UV CD in the absence and presence of ozone in phosphate buffer and diluted versions with different water types were examined and compared in Figure 3. Changes seen in the Hb Far-UV CD in the absence (Figure 3A) and presence of ozone at two different concentrations of 55 and 80 μg/ml (Figures 3B,C, respectively) are shown.

Far-UV CD spectra of Hb samples in the absence of ozone in undiluted phosphate buffer and diluted versions with deionized and tap water did not show much change in the secondary structure content of Hb. It was only in the presence of added double distilled water that the intensity of the peaks relative to undiluted phosphate buffer showed a decrease in alpha helix content and an almost unwanted irregular structure. Having said that, CD spectra of Hb samples in undiluted phosphate buffer and diluted versions with deionized, double distilled and tap water and in the presence of ozone at concentrations of 55 and 80 μg/ml, showed no significant changes in the intensity of the peaks.

In Figures 3D–G, Hb samples in undiluted phosphate buffer and diluted versions with deionized, double distilled and tap water were analyzed in the absence and presence of ozone at 55 and 80 μg/ml. In the three cases (Figures 3D,E,G), when ozonated Hb were analyzed in phosphate buffer, deionized water and tap water, no changes were observed for the peak intensities in comparison to non-ozonated Hb control samples. However, in the presence of double distilled water (Figure 3F), the non-ozonated Hb control sample was shown to have a decrease in peak intensity of the two peaks compared to the ozonated samples of Hb in the presence of added double distilled water. In Figures 3H,I, the Far-UV CD results for Hb samples in undiluted phosphate buffer and diluted versions with deionized, double distilled and tap water were further compared at 55 and 80 μg/ml ozone. The results confirmed that the major changes (the undesirable reduction in peak intensity) was related to the non-ozonated Hb sample when phosphate buffer was diluted with double distilled water.

#### Near-UV CD

Near-UV CD spectra shows changes in heme binding to globin and spin state changes of iron atom. These alterations are visible through the strongly positive peak at a wavelength of 260 nm, also called the L band. The decrease in this peak intensity means that the heme binding to the globin is weakened and the spin state of the iron atom is altered (Gabbianelli et al., 2004; Nagai et al., 2014). Near-UV also shows changes in aromatic amino acids, especially tryptophan in the globular structure of the protein, at a wavelength of about 290 nm (Li et al., 2000). The changes are less evident than the L-band.

**FIGURE 4:**
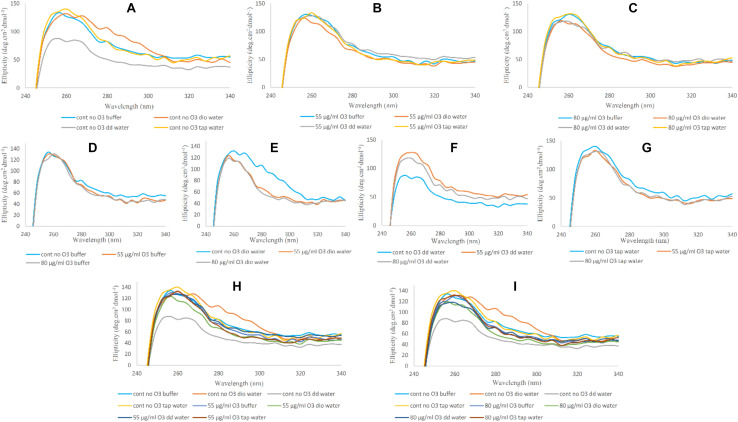
Near-UV CD spectra of non-ozonated and ozonated Hb samples in the presence of phosphate buffer and diluted versions with different water types. **(A)** Non-ozonated Hb in phosphate buffer and different water types. **(B)** Hb ozonated with 55 μg/ml ozone in phosphate buffer and different water types. **(C)** Hb ozonated with 80 μg/ml ozone in phosphate buffer and different water types. **(D)** Non-ozonated and ozonated Hb with 55 and 80 μg/ml ozone in phosphate buffer. **(E)** Non-ozonated and ozonated Hb with 55 and 80 μg/ml ozone in phosphate buffer diluted with deionized (Dio) Water. **(F)** Non-ozonated and ozonated Hb with 55 and 80 μg/ml ozone in phosphate buffer diluted with double distilled (DD) Water. **(G)** Non-ozonated and ozonated Hb with 55 and 80 μg/ml ozone in phosphate buffer diluted with Tap Water. **(H)** Non-ozonated and ozonated Hb with 55 μg/ml ozone in phosphate buffer and diluted in various different water types. **(I)** Non-ozonated and ozonated Hb with 80 μg/ml ozone in phosphate buffer and diluted in various different water types.

In Figure 4, variations in the spectra of Near-UV for Hb in undiluted phosphate buffer and diluted versions with deionized, double distilled and tap water in the absence and presence of ozone are shown. In Figure 4A, we see the greatest variations in the peak intensity at 260 nm in the non-ozonated Hb samples in the presence of different waters. The lowest peak intensity was related to the non-ozonated Hb sample in the presence of phosphate buffer diluted with double distilled water, in agreement with far-UV CD results. Hb samples in the presence of phosphate buffer, diluted versions with deionized water and tap water showed the same peak intensities. However, the peak intensity of Hb in the presence of deionized water shifted to longer wavelengths. In Hb samples ozonated with 55 and 80 μg/ml (Figures 4B,C, respectively), no significant changes were observed in the spectra of the Near-UV. Only the Hb sample ozonated in the presence of deionized water showed a slight decrease in peak intensity. In Figures 4D–G, we investigated whether the peak changes of Hb samples in undiluted phosphate buffer and diluted versions with deionized, double distilled and tap water were due to the absence or presence of ozone. Results showed that the peak changes were seen to be more related to non-ozonated Hb samples in phosphate buffer diluted with deionized and double distilled water types (Figures 4E,F, respectively). Therefore, in the presence of deionized water (Figure 4E), the non-ozonated Hb sample showed a higher peak intensity and shifted to the right compared to the two ozonated samples, and there was no difference between the two ozonated Hb samples in deionized water. However, in the presence of double distilled water (Figure 4F), the non-ozonated Hb sample showed a sharp decrease in peak intensity compared to the two ozonated samples, while the two ozonated samples did not differ significantly. In Figures 4H,I, the general changes in peak intensities of Hb samples in the presence of phosphate buffer and diluted versions with different water types, are shown by comparing the non-ozonated samples with that of ozonated samples at 55 and 80 μg/ml ozone.

#### Soret-UV CD

**FIGURE 5:**
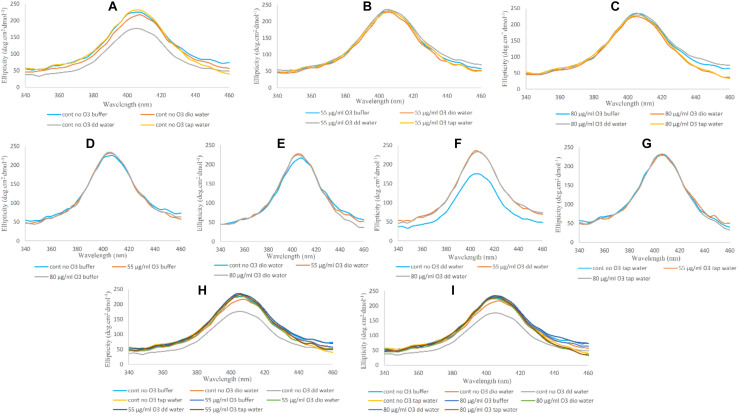
Soret-UV CD spectra of non-ozonated and ozonated Hb samples in the presence of phosphate buffer and diluted versions with different water types. **(A)** Non-ozonated Hb in phosphate buffer and different water types. **(B)** Hb ozonated with 55 μg/ml ozone in phosphate buffer and different water types. **(C)** Hb ozonated with 80 μg/ml ozone in phosphate buffer and different water types. **(D)** Non-ozonated and ozonated Hb with 55 and 80 μg/ml ozone in phosphate buffer. **(E)** Non-ozonated and ozonated Hb with 55 and 80 μg/ml ozone in phosphate buffer diluted with deionized (Dio) Water. **(F)** Non-ozonated and ozonated Hb with 55 and 80 μg/ml ozone in phosphate buffer diluted with double distilled (DD) Water. **(G)** Non-ozonated and ozonated Hb with 55 and 80 μg/ml ozone in phosphate buffer diluted with Tap Water. **(H)** Non-ozonated and ozonated Hb with 55 μg/ml ozone in phosphate buffer and diluted in various different water types. **(I)** Non-ozonated and ozonated Hb with 80 μg/ml ozone in phosphate buffer and diluted in various different water types.

Binding of the heme group to the globin and oxygen binding to the heme in CD spectra is characterized by a marked positive peak at 414 nm, which is called Soret- or B-band (Munoz and De Juan, 2007). Reduction in peak intensity of the Soret-UV band is indicative of alterations in the heme prosthetic group and weakening of heme binding to globin compared to its native form. In Figure 5, changes in the Soret band and heme group are shown. In Figures 5A–C, the only change seen was related to the Hb sample in the presence of double distilled water, showing a decrease in band intensity, consistent with Far-UV and Near-UV CD results. However, the ozonated Hb samples did not show any significant changes compared to the non-ozonated Hb samples. In Figures 5D–G, the effect of the different water types on Hb in the absence and presence of ozone on the Soret band of Hb are shown. Non-ozonated Hb in the presence of double distilled water (Figure 5F), showed a reduction in the intensity of the Soret band compared to the ozonated Hb samples of the same water type. In Figures 5H,I, the general changes in band intensity of Hb samples in the presence of different waters are shown by comparing non-ozonated samples with that of ozonated samples at 55 and 80 μg/ml ozone.

### UV-VIS Absorption Spectroscopy

**FIGURE 6:**
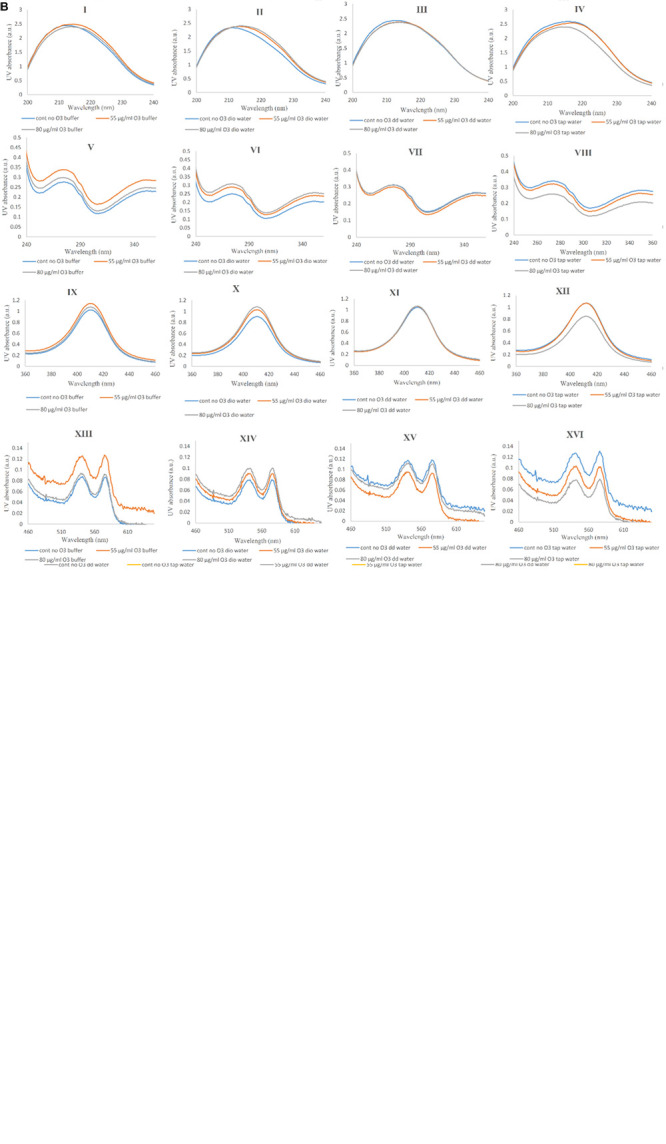
UV-Vis spectra of non-ozonated and ozonated Hb samples in the presence of phosphate buffer and diluted versions with different water types. **(A)** UV-Vis spectra comparing Hb in phosphate buffer with different water types in the presence and absence of two different concentrations of ozone. (I, II, III) Non-ozonated, Hb ozonated at 55 and 80 μg/ml in phosphate buffer and different water types, respectively (wavelength range 200–240 nm). (IV, V, VI) Non-ozonated, Hb ozonated at 55 and 80 μg/ml in phosphate buffer and different water types, respectively (wavelength range 240–360 nm). (VII, VIII, IX) Non-ozonated, Hb ozonated at 55 and 80 μg/ml in phosphate buffer and different water types, respectively (wavelength range 360–460 nm). (X, XI, XII) Non-ozonated, Hb ozonated at 55 and 80 μg/ml in phosphate buffer and different water types, respectively (wavelength range 460–660 nm). **(B)** UV-Vis spectra comparing the effects of two different ozone concentrations on Hb in the different solvents including phosphate buffer and the diluted versions of it with different water types. (I, II, III, IV) Non-ozonated, Hb ozonated at 55 and 80 μg/ml ozone in phosphate buffer, Dio water, DD water and tap water, receptively (wavelength range 200–240 nm). (V, VI, VII, VIII) Non-ozonated, Hb ozonated at 55 and 80 μg/ml ozone in phosphate buffer, deionized (Dio) water, double distilled (DD) water and tap water, respectively (wavelength range 240–360 nm). (IX, X, XI, XII) Non-ozonated, Hb ozonated at 55 and 80 μg/ml ozone in phosphate buffer, Dio water, DD water and tap water, receptively (wavelength range 360–460 nm). (XIII, XIV, XV, XVI) Non-ozonated, Hb ozonated at 55 and 80 μg/ml ozone in phosphate buffer, Dio water, DD water and tap water, respectively (wavelength range 460–660 nm). In both panels **(A)** and **(B)**, the first, second, third, and fourth rows indicate changes in peptide bonds, aromatic residues, B-band and Q-bands, respectively.

In general, changes in the secondary and tertiary structures of a protein, as well as the binding of heme to heme-containing protein can be studied by UV-VIS absorption spectroscopy (Munoz and De Juan, 2007). These changes are identified through the following peaks: 222 nm as a result of n-π^∗^ transition of amidic/peptide bonds, and 278 nm due to aromatic residues including tryptophan, tyrosine and phenylalanine (Sharma and Graham, 2010). The π electrons in the porphyrin ring at the UV-VIS spectrum show three distinct peaks: One at 414 nm called the Soret or B-band like a Soret-CD, and another pair of bands at longer wavelengths (542 and 577 nm) in the visible region (Cataldo and Gentilini, 2005). In Figure 6A, the variations in the Hb UV-VIS spectra of Hb samples are shown in panels I to XII (panels I, IV, VII and X show results of samples without ozone; panels II, V, VIII and XI show results of samples ozonated at 55 μg/ml; and panels III, VI, IX and XII show results of samples ozonated at 80 μg/ml) in the presence of different water types. In panels I, II and III, changes in peptide bonds are negligible in all three cases and only the sample of non-ozonated Hb diluted with tap water (panel I), shifted slightly towards the longer wavelength (red shift).

In panels IV, V and VI and in the case of non-ozonated Hb samples (in panel IV), the maximum peak intensity was related to the sample in the presence of tap water and the lowest peak intensity belonged to the sample of Hb in the presence of deionized water. Peaks of Hb samples without ozone and in the presence of phosphate buffer and further diluted samples with double distilled water were almost the same. Changes in aromatic residues are less evident in samples of ozonated Hb with 55 μg/ml ozone and most of the samples, unlike phosphate buffer, showed a slight reduction in peak intensity in the presence of different water types (panel V). Hb ozonated at 80 μg/ml in the presence of tap water, showed a decrease in peak intensity compared to the rest of the samples (panel VI). Therefore, the sample of Hb in the presence of tap water without ozone showed an increase in peak intensity, whereas ozonation with 50 μg/ml and especially 80 μg/ml ozone, reduced the peak intensity associated with the aromatic residues. Additionally, the increase in the intensity of the Soret band (panels VII, VIII and IX) and Q-bands (panels X, XI and XII) in the non-ozonated samples of Hb in the presence of tap water was comparable with a reduction in peak intensity in Hb sample in the presence of deionized water. However, upon ozonation of Hb in tap water, these peak intensities were reduced, especially when using 80 μg/ml. In non-ozonated and ozonated Hb samples at 80 μg/ml, contrary to 55 μg/ml, we find that the sample of Hb in the presence of double distilled water showed a higher peak intensity compared to Hb in the undiluted phosphate buffer (panels X, XI and XII). Figure 6B compares the UV-Vis spectra of the Hb samples in the presence of phosphate buffer (panels I, V, IX and XIII) and diluted versions with deionized water (panels II, VI, X and XIV), double distilled water (panels III, VII, XI and XV) and tap water (panels IV, VIII, XII and XVI) in non-ozonated and ozonated states. In panels I, II, III and IV, changes in the peptide band of Hb in the presence of deionized water (panel II) and tap water (panel IV) are slightly different from that of the other two waters such that the Hb sample in the presence of deionized water ozonated at both concentrations of ozone, showed a peak shift (red shift) compared to non-ozonated Hb in deionized water. However, in the presence of tap water, Hb ozonated with 80 μg/ml ozone (panel IV) caused reduction in the intensity of the peptide band compared to Hb samples in the other two water types. In panels V, VI, VII and VIII changes related to aromatic residues are shown. Ozonation of Hb, especially at 55 μg/ml ozone in phosphate buffer, increased the peak intensity associated with aromatic residues. This was also the case for Hb in phosphate buffer diluted with deionized water, but there was no significant difference between the two concentrations of ozone. Changes in aromatice residues in the presence of double distilled water were not significantly different in the presence and absence of ozone. Only in the presence of tap water, the severity of the peak was reduced upon ozonation, especially at 80 μg/ml ozone, relative to the non-ozonated sample. In panels IX, X, XI and XII, the changes in the heme group is shown. Ozonated Hb in the presence of phosphate buffer and deionized water, increased the intensity of the peak related to heme. As for double distilled water, no changes were detected for Hb samples in the presence or absence of ozone. However, the sample of Hb diluted with tap water and ozonated at 80 μg/ml resulted in a significant reduction in the Soret band and a possible reduction in oxygen binding to heme. Q-band changes are shown in panels XIII, XIV, XV and XVI. Overall, all Hb samples were shown to be in their oxygenated state as identified through the existence of two distinguishable peaks at 542 and 577 nm. However, ozonated Hb samples in the presence of phosphate buffer and those diluted with deionized water increased the intensity of the Q-bands, while Hb samples diluted with double distilled water and tap water, in particular, caused a reduction in the band intensity. In the presence of phosphate buffer and double distilled water, ozonated Hb at 80 μg/ml ozone showed a band intensity close to the non-ozonated Hb sample, and only ozonation at 55 μg/ml caused a large change such that an increase in the intensity of the Q-bands was observed for Hb sample in phosphate buffer and a decrease in Hb sample diluted with double distilled water. The changes in the intensity of the Q-bands are related to the alterations in porphyrin rings detectable from their π electrons.

Therefore, ozonated Hb (at 80 μg/ml) in phosphate buffer and diluted version with double distilled water did not show changes in the Q-bands, while ozonated Hb at the same ozone concentration in the presence of deionized water and tap water caused the most significant changes such that an increase in the intensity of Q-bands was observed for Hb in the presence of deionized water and a decrease in the presence of tap water.

### SDS- and Native-PAGE

Hb is a macromolecular tetramer consisting of four polypeptide chains that are linked through non-covalent bonds and each chain contains a heme group (Stryer, 1995). In SDS-PAGE, protein movement is strongly dependent on features such as being hydrodynamic, shape and level of surface charge (Carvalho et al., 2011).

**FIGURE 7:**
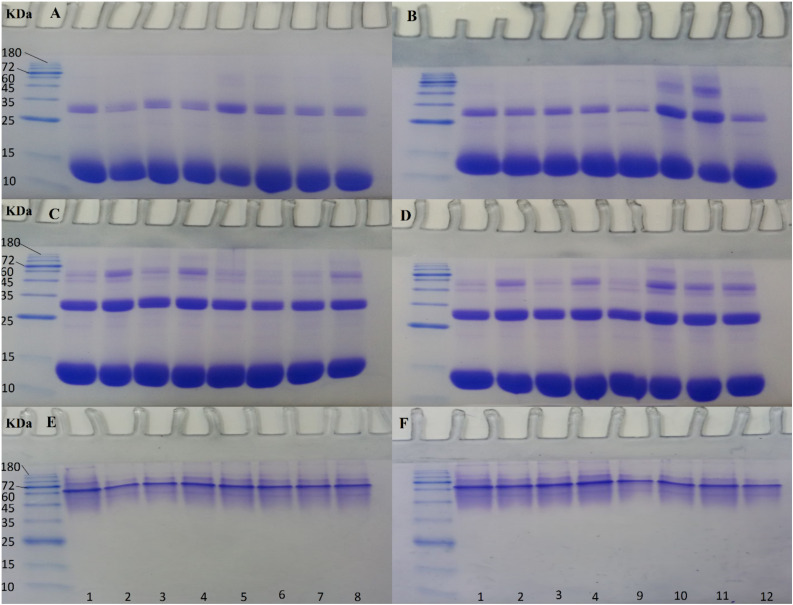
SDS- and Native-PAGE analyses of non-ozonated and ozonated Hb samples in the presence of phosphate buffer and diluted versions with different water types. Samples 1 to 4: non-ozonated Hb samples in phosphate buffer, deionized water, double distilled water, and tap water, respectively. Samples 5 to 8: ozonated Hb samples at 55 μg/ml in phosphate buffer, deionized water, double distilled water, and tap water, respectively. Samples 9 to 12: ozonated Hb samples at 80 μg/ml in phosphate buffer, deionized water, double distilled water, and tap water, respectively. The samples in SDS-PAGE gels **(A)** and **(B)** are in the presence of DTT and in gels **(C)** and **(D)** in the absence of DTT. Samples in Native-PAGE gels **(E)** and **(F)** are under native conditions.

As in our previous work (Mehraban et al., 2018), DTT was used to eliminate disulfide bonds, so in the presence of DTT (Figures 7A,B), the main thick protein band between 10 and 15 kDa marker bands, was indicative of the reduced and denatured globins of Hb with almost similar molecular weights. The next obvious protein band with less intensity belonged to the Hb dimer band (about 32 kDa) between the 25 and 35 kDa marker bands. In the presence of DTT, there were additional bands almost near and above the 45 kDa marker band, which may be present either due to the degradation of Hb by ROS randomly attacking the carbon methene bonds in the tetrapyrrole rings and creating various pyrrole products that can bind together by covalent bonds, as reported previously (Nagababu and Rifkind, 2004) or be due to the formation of trimers and tetramers of Hb through the formation of covalent di-tyrosine cross-links (Kampf et al., 2015). The higher molecular weight bands were not detected in non-ozonated Hb samples using SDS-PAGE in the presence of DTT, while it was observed clearly in Hb samples ozonated with 55 μg/ml ozone in phosphate buffer and with less intensity in the presence of deionized water (Figure 7A, lanes 5 and 6) and in samples of Hb ozonated at 80 μg/ml in the presence of deionized water, and in particular, double-distilled water (Figure 7B, lanes 10 and 11). As such, these samples revealed slightly lower intensity for the monomeric bandwidth and instead had a slightly more intense dimer bandwidth and possibly trimers, tetramers and oligomers. Comparing non-ozonated Hb sample in phosphate buffer with ozonated Hb samples diluted with various water types (Figures 7A,B), it was observable that the higher concentration of ozone (80 μg/ml) had a greater effect on increasing the dimer bandwidth or forming trimeric, tetrameric and oligomeric forms of Hb (Figure 7B, lanes 10 and 11).

In the absence of DTT, however (Figures 7C,D), the possibility of forming a smear of additional bands including trimers, tetramers and oligomers due to the formation of both di-sulfide bonds and covalent bonds, was more significant for the non-ozonated control Hb samples in the presence of deionized and tap water (Figure 7C, lanes 2 and 4, respectively), for ozonated Hb samples at 55 μg/ml in the presence of tap water followed by the undiluted phosphate buffer (Figure 7C, lanes 8 and 5, respectively), and for ozonated Hb samples at 80 μg/ml in the presence of deionized water followed by double distilled and tap water (Figure 7D, lanes 10, 11 and 12, respectively). In the absence of DTT, when comparing non-ozonated and ozonated Hb samples in the undiluted phosphate buffer (Figures 7C,D, lanes 1, 5 and 9), the lowest probability of forming larger molecular weight bands was related to the sample of non-ozonated Hb. It is worth mentioning that there were no significant changes observed in the formation of higher molecular weight bands in either the non-ozonated or ozonated Hb samples prepared in phosphate buffer or its diluted version with tap water. However, the main changes were observed for Hb samples diluted with deionized water, in particular, followed by double distilled water ozonated at 80 μg/ml.

Native-PAGE for non-ozonated and ozonated Hb samples in the presence of phosphate buffer and diluted versions with different water types confirmed the presence of the Hb tetrameric band between the 60 and 72 kDa protein marker bands.

### Dynamic Light Scattering

**FIGURE 8:**
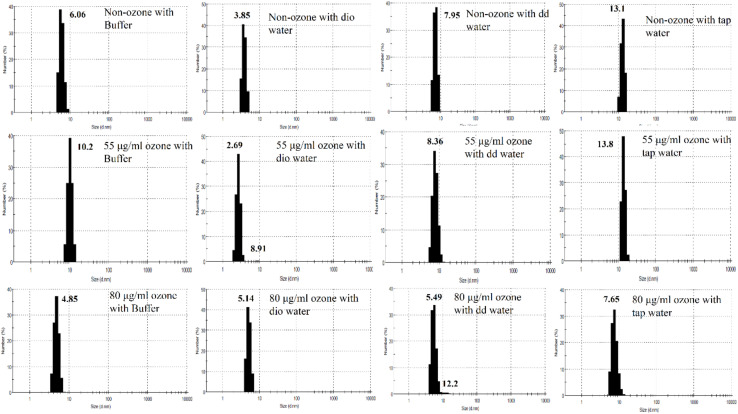
Diameter size distribution of non-ozonated and ozonated Hb samples in the presence of phosphate buffer and diluted versions with different water types dio and dd water refer to deionized and double distilled water, respectively.

DLS results of non-ozonated and ozonated Hb at 55 and 80 μg/ml in phosphate buffer and diluted versions with various water types are given in Figure 8 and Table 3. It should be emphasized that information obtained from DLS in both the number and intensity modes as well as the polydispersity index (PDI) values are to be considered together when analyzing the data from this technique. The non-ozonated Hb sample in phosphate buffer had a diameter size of 6.06 nm, which was reduced in the presence of deionized water to 3.85 nm, but with the largest diameter size of 568 nm (93.1% composition) compared to 304 nm (93.6% composition) in the intensity mode. As for non-ozonated Hb samples in the presence of double distilled and tap water, the diameter sizes were increased to 7.95 nm and 13.1 nm, respectively, in the number mode (however, corresponding to a diameter size of 368 nm and 103 nm at 51.7% and 93.4% composition, respectively). These findings are in line with the results of SDS-PAGE (Figure 7C, lanes 2 and 4), especially for non-ozonated Hb samples diluted with deionized water and tap water (with reference to data in the intensity and number modes, respectively), indicating the presence of additional higher molecular weight bands, potentially due to degradation caused by ROS randomly attacking the carbon methene bonds in the tetrapyrrole rings and forming different pyrrole products through covalent bonds or trimers or tetramers through covalent di-tyrosine cross-links, and in particular disulfide bonds in the absence of DTT. PDI values also showed the highest value for tap water at 0.662.

**TABLE 3:** Size distribution analysis of Hb samples in number and intensity modes as well as percentage intensity and PDI values using DLS. Dio and Dd water refer to deionized and double distilled water, respectively.

Samples	Ozone concentrations	Diameter (nm) (number mode)	Diameter (nm) (intensity mode)	Intensity% (intensity mode)	PDI
Oxy-Hb buffer	Non-ozone	6.06	6.38	6.4	0.519
			304	93.6	
Oxy-Hb Dio water	Non-ozone	3.85	3.96	6.9	0.481
			568	93.1	
Oxy-Hb Dd water	Non-ozone	7.95	7.26	31.3	0.532
			83.2	16.9	
			368	51.7	
Oxy-Hb Tap water	Non-ozone	13.1	13.3	6.6	0.662
			103	93.4	
Oxy-Hb Buffer	55 μg/ml	10.2	10.7	5.9	0.540
			375	94.1	
Oxy-Hb Dio water	55 μg/ml	2.69	2.77	5.4	0.416
		8.91	9.46	30.2	
			46.8	64.4	
Oxy-Hb Dd water	55 μg/ml	8.36	8.82	27.6	0.230
			44.8	72.4	
Oxy-Hb Tap water	55 μg/ml	13.8	13.9	7.2	0.318
			127	92.8	0.318
Oxy-Hb Buffer	80 μg/ml	4.85	5.17	24	0.778
			27.7	8.3	
			237	67.7	
Oxy-Hb Dio water	80 μg/ml	5.14	5.28	7	0.994
			178	93	
Oxy-Hb Dd water	80 μg/ml	5.49	6.02	44	0.493
		12.2	13.2	56	
Oxy-Hb Tap water	80 μg/ml	7.65	8.97	10.3	0.946
			214	84.8	
			4860	4.9	

*Dio and Dd water refer to deionized and double distilled water, respectively.*

Results of Hb samples ozonated with 55 μg/ml ozone in phosphate buffer and diluted versions with various water types revealed that the Hb diameter size was largest in the presence of tap water, followed by the undiluted phosphate buffer, double distilled and deionized water, in the number mode. This was consistent with the results of SDS-PAGE (Figure 7C, lanes 5–8). Additional higher molecular weight species of Hb possibly formed through disulfide and covalent bonds were observed. The highest PDI values, however, were related to the Hb samples in the presence of undiluted phosphate buffer followed by deionized water instead of tap water (Table 3). These effects can also be seen to some extent in the SDS-PAGE results in the presence of DTT (Figure 7A).

Results of Hb samples ozonated with 80 μg/ml ozone showed that the smallest diameter size was related to the Hb sample in phosphate buffer, where the dimeric bandwidth was reduced greatly in SDS-PAGE, especially in the presence of DTT (Figure 7B). As for Hb samples ozonated in double distilled and deionized water, with a collective consideration of the data obtained from DLS, it can be deduced that there was increased oligomerization, in line with the significant presence of higher molecular weight bands seen in SDS-PAGE results (Figure 7B, lanes 10 and 11). In the absence of DTT (Figure 7D), the same observation was detected mainly in Hb samples in deionized water, followed by both double distilled and tap water. The highest PDI value also belonged to Hb in deionized water followed by tap water, which is in line with results of SDS-PAGE (Figure 7D).

## Discussion

It is worth mentioning that with regards to our previous work (Mehraban et al., 2018), when purified Hb in the absence of antioxidant was treated directly with ozone bubbles (method B in our previous study), a significant decrease in the red color of Hb was observed, as a sign of heme breakdown. However, in this study, purified Hb in the absence of antioxidants was ozonated in a syringe, as in method A, at a volumetric ratio of 1 to 1 (Hb: ozone), and no major color change was observed (Figure 1). In fact, even though the presence of antioxidants are important but the safe method for ozone therapy or autohemotherapy is method A and not the direct use of ozone gas bubbles (method B). In addition to identifying method A as the safe method for autohemotherapy, we also investigated in this study, the effect of pressure produced in the syringe based on its volumetric size. This revealed very important and critical information showing that increasing the pressure in the syringe, by reducing the volumetric size of the syringe from 10 to 5 ml, in the process of ozonation of Hb in phosphate buffer and diluted versions using various water types, resulted in significant and undesirable Hb precipitation.

As for the significant importance of antioxidants, DLS results from this study showed that the diameter size of purified Hb (in the absence of antioxidant) ozonated at 55 μg/ml in undiluted phosphate buffer was 10.2 nm whereas the diameter size of the Hb sample of the same individual ozonated at the same concentration in phosphate buffer and in the presence of antioxidants as shown in our other work (Mehraban et al., 2019) was 6.07 nm. This increase in the size of the Hb diameter in the presence of the same buffer and the same ozone concentration can be due to the absence of antioxidants and hence the increase in the amount of oxidation of Hb resulting in increasing amounts of di-sulfide bonding (Carvalho et al., 2011) or covalent di-tyrosine cross-linking (Kampf et al., 2015).

Analysis of the different water types used in this study revealed the existence of a number of ions including bicarbonate ions (defined as water alkalinity), calcium, chloride and sulfate ions in tap water. Neither deionized nor double distilled water contained any ions, while phosphate buffer contained phosphate ions solely. The dilution of phosphate buffer from 50 to 10 mM, with ion and non-ion containing water types, both in the presence and absence of ozone, showed no significant pH changes. This indicated that phosphate buffer diluted to 10 mM could adequately maintain the pH and was not the major cause of changes seen when comparing the undiluted and diluted Hb samples. In actual fact, the presence or absence of ions seem to be the major contributory factor for to the changes observed with regards to Hb structure and its oligormerisation. The molar ionic conductivity of each ionic species is proportional to its electrical mobility (Laidler and Meiser, 1982). Thus, due to the presence of different ions in tap water and phosphate buffer, different EC values were obtained. The effects of ions on the conformation and function of protein have been investigated and it has been shown that conformational changes in protein in the presence of ions can be mainly through ionic effects on water structure (Damodaran and Kinsella, 1982).

In general and from the fluorescence spectral analysis, Hb samples ozonated at the two different ozone concentrations compared to non-ozonated Hb samples showed a reduction in peak intensity, which can be due to changes and a greater polarization in the surrounding environment of the fluorophores resulting in a more polar and more open structure. In Hb samples diluted with tap water, at both ozone concentrations, no effect was detected by the fluorescence spectra compared to the non-ozoned state of Hb, which can be due to the presence of ions in this water type. This effect was not seen in the two other water types (deionized and double distilled waters), due to the absence of ions that otherwise exist in tap water. With regards to Far-UV CD data, only in non-ozonated Hb samples in phosphate buffer diluted with double distilled water, a decrease in alpha helix content and an almost unwanted irregular structure was observed compared to ozonated samples at the two different concentrations and phosphate buffer diluted with other water types, which did not show any significant changes in the secondary structure of Hb. Furthermore, data from Near-UV and Soret CD, showed significant changes in the tertiary structure and possibility of weakening the heme binding to the globin only in the non-ozonated Hb sample diluted with double distilled water. However, changes in the tertiary structure and shift to longer wavelengths were also observed in the non-ozonated sample of Hb diluted with deionized water. Looking at the UV spectra of ozonated Hb samples, especially at 80 μg/ml ozone, and at the non-ozonated samples, the major changes were related to the Hb sample diluted with tap water, with an increase in intensity in the non-ozonated Hb sample and a decrease in the presence of ozone at 80 μg/ml. This implied that the presence of ions in tap water can affect the intensity of peaks detected from the porphyrin rings of Hb via changes in π electrons.

Based on the results from SDS-PAGE and DLS, comparing the diameter size of non-ozonated and ozonated Hb samples diluted with different water types, showed that the presence of phosphate ions at 50 mM is very important in maintaining the diameter size of Hb, such that by looking at Figure 8 and Table 3, we can see the increasing Hb diameter size in samples diluted with different water types as compared to the undiluted phosphate buffer in terms of number and intensity modes and PDI values. This result is also consistent with that observed in Figure 7. The results further show the highest increase in the diameter size corresponding to the non-ozonated Hb sample diluted with tap water. This increase is also observed in Hb samples ozonated with 55 and 80 μg/ml ozone diluted with different water types, especially tap water. However, at the concentration of 80 μg/ml ozone, the highest PDI value was related to the Hb sample diluted with deionized water and then tap water, but the highest diameter size of Hb in the number mode belonged to the Hb sample diluted with double distilled water with two peaks (5.49 and 12.2 nm), consistent with the SDS-PAGE results (Figures 7B,D). With regards to tap water, while the existence of calcium, chloride and sulfate ions seemed to cause an overall increased level of oligomerization and diameter size (based on SDS-PAGE and DLS results) compared to the other Hb samples in this study, the presence of bicarbonate ions acted to scavenge OH radicals and reduced the level of Hb oxidation and oligomerization at higher ozone concentrations, similar to the results seen for Hb samples in undiluted phosphate buffer, due to the presence of an adequate concentration of phosphate ions.

Therefore, the presence of phosphate and bicarbonate ions at higher concentrations of ozone showed to reduce the amount of Hb oxidation and hence prevented the formation of disulfide or covalent di-tyrosine bonds. This effect was not seen in other water types due to the absence of these ions, thus resulting in oligomerization of Hb (Figure 7B, lanes 10 and 11).

## Conclusion

Comparing all the data and in particular SDS-PAGE results under reducing conditions (Figures 7B,D), it can be concluded that the concentration of 50 mM phosphate buffer was necessary to prevent the unfavorable effects of Hb oxidation imposed by ozone at high concentrations. Additionally, Hb diluted with tap water was also able to maintain relative consistency in response to ozone at high concentrations due to its alkalinity or presence of bicarbonate ions, which is known to help scavenge OH radicals and prevent superoxide formation. Having said that, other ions in tap water including calcium, chloride and sulfate, work in the opposite direction and have unfavorable effects in terms of Hb oligomerization. Non-ion containing deionized and double distilled waters, with only 10 mM phosphate buffer, did not show the ability to withstand the oxidation pressures when exposed to high concentrations of ozone and clearly showed the damage done through oligomerization of Hb. Therefore, it can be concluded from this study that the presence of ions such as phosphate and bicarbonate, in adequate amounts, can withstand the unfavorable effects of oxidation and oligomerization of Hb upon ozonation at high ozone concentrations, which is an important finding to be considered in autohemotherapy.

## Data Availability Statement

All datasets generated for this study are included in the article.

## Ethics Statement

The use of human blood sample in this study was reviewed and approved by ethics code IR.UT.SPORT.REC.1397.038 given by the Iran National Committee for Ethics in Biomedical Research. The ethics committee meeting was conducted at the Faculty of Physical Education and Sports Sciences, University of Tehran. Written informed consent for participation was not required for this study in accordance with the national legislation and the institutional requirements.

## Author Contributions

FM: investigation, formal analysis, methodology, writing of the original draft, and arranged for sample collection. AS: conceptualization, investigation, formal analysis, methodology, funding, project administration, resources, supervision, and writing – review. SR: contribution to the manuscript by performing and analyzing the conductivity measurements of the different water types. VM: producer and provider of the Gardina ozone machine who also provided funding for the project. All authors read and approved the final manuscript.

## Conflict of Interest

The authors declare that the research was conducted in the absence of any commercial or financial relationships that could be construed as a potential conflict of interest.

## Abbreviations

Buffer: phosphate buffer
Dd water: double distilled water
Dio water, deionized water;Hb: hemoglobin.
